# Synergy of Electrostatic and Chemical Doping to Improve the Performance of Junctionless Carbon Nanotube Tunneling Field-Effect Transistors: Ultrascaling, Energy-Efficiency, and High Switching Performance

**DOI:** 10.3390/nano12030462

**Published:** 2022-01-28

**Authors:** Khalil Tamersit, Abdellah Kouzou, Hocine Bourouba, Ralph Kennel, Mohamed Abdelrahem

**Affiliations:** 1 Department of Electronics and Telecommunications, Université 8 Mai 1945 Guelma, Guelma 24000, Algeria; bourouba.hocine@univ-guelma.dz; 2 Department of Electrical and Automatic Engineering, Université 8 Mai 1945 Guelma, Guelma 24000, Algeria; 3 Laboratory of Inverse Problems, Modeling, Information and Systems (PIMIS), Université 8 Mai 1945 Guelma, Guelma 24000, Algeria; 4 Applied Automation and Industrial Diagnosis Laboratory (LAADI), Faculty of Science and Technology, Djelfa University, Djelfa 17000, Algeria; kouzouabdellah@ieee.org; 5 Electrical and Electronics Engineering Department, Nisantasi University, Istanbul 34398, Turkey; 6 Institute for Electrical Drive Systems and Power Electronics (EAL), Technical University of Munich (TUM), 80333 Munich, Germany; ralph.kennel@tum.de; 7 Electrical Engineering Department, Faculty of Engineering, Assiut University, Assiut 71516, Egypt

**Keywords:** carbon nanotube, junctionless, tunnel field effect transistors, chemical doping, electrostatic doping, NEGF simulation, band-to-band tunneling, switching performance, nanoscale

## Abstract

The low on-current and direct source-to-drain tunneling (DSDT) issues are the main drawbacks in the ultrascaled tunneling field-effect transistors based on carbon nanotube and ribbons. In this article, the performance of nanoscale junctionless carbon nanotube tunneling field-effect transistors (JL CNTTFETs) is greatly improved by using the synergy of electrostatic and chemical doping engineering. The computational investigation is conducted via a quantum simulation approach, which solves self-consistently the Poisson equation and the non-equilibrium Green’s function (NEGF) formalism in the ballistic limit. The proposed high-performance JL CNTTFET is endowed with a particular doping approach in the aim of shrinking the band-to-band tunneling (BTBT) window and dilating the direct source-to-drain tunneling window, while keeping the junctionless paradigm. The obtained improvements include the on-current, off-current, ambipolar behavior, leakage current, I_60_ metric, subthreshold swing, current ratio, intrinsic delay, and power-delay product. The scaling capability of the proposed design was also assessed, where greatly improved switching performance and sub-thermionic subthreshold swing were recorded by using JL CNTTFET with 5 nm gate length. Moreover, a ferroelectric-based gating approach was employed for more enhancements, where further improvements in terms of switching performance were recorded. The obtained results and the conducted quantum transport analyses indicate that the proposed improvement approach can be followed to improve similar cutting-edge ultrascaled junctionless tunnel field-effect transistors based on emerging atomically thin nanomaterials.

## 1. Introduction

Sub-thermionic subthreshold swing provided by nanoscale-tunnel field-effect transistors (TFETs) enables a decrease in power supply voltage, which is a prerequisite in ultralow power applications, such as the internet of things (IoT) [1,2]. In the last decade, the great progress experienced in nanomaterials science have given an additional asset and new impulses to TFETs technology, which can play a leading role in the extension of Moore’s Law that converges to its end [2,3,4,5]. In the ultrascaled regime, the accuracy of nanofabrication is crucial for the reliability of elementary nanoelectronic nanodevices, as it can affect the performance of electronic circuits and systems [2,3,4,5,6]. In this context, the junctionless paradigm has shown its efficiency in simplifying the elaboration of ultrascaled FETs on the one hand and in improving their performance on the other hand [6,7,8,9]. Combining the benefits of the junctionless paradigm with the amazing features of tunneling transistors has been the subject of promising devices called junctionless-tunnel field-effect transistors (JL TFETs) [9]. In these devices, the channel doping is performed by the electrostatic and chemical doping, while ensuring the operating regime of tunneling FETs with a double benefit in terms of the facility of fabrication and high performance [9,10].

In the literature, the junctionless-carbon-nanotube-tunnel field-effect transistors (JL CNTTFETs) have shown promising subthreshold and switching performance [5,10,11] due to the amazing characteristics of carbon nanotube (CNT) as mature channel material, such as atomic structure, tunable band gap, high electrical conductivity, quasi-ballistic property, high Fermi velocity, and high sensitivity to its surrounded electrostatics (i.e., the electrostatic gating) [12,13,14,15]. However, as any electronic nanodevice, the ultrascaled JL CNTTFET suffers from some weaknesses, namely the low on-current and the issue of direct source-to-drain tunneling (DSDT), which is attributed to the low effective mass in the carbon nanotube [16,17,18]. Note that the DSDT phenomenon is the main cause in degrading the switching and subthreshold performance of CNT tunnel FETs in ultrascaled regime [18]. Recently, an ultrascaled CNTTFET with p-n doping profile has been proposed, showing spectacular improvements in terms of subthreshold and switching performance, including the on-current [18]. However, the p-n junction is still an intractable task even with the experienced progress in nanofabrication, including the doping techniques. A negative-capacitance carbon-nanotube-tunnel field-effect transistor (NC CNTTFET) has also been proposed recently through a quantum simulation study, where improved on-current was recorded by dint of ferroelectric-induced amplified inner-gate voltage [19]. However, the instability in NC-FET is still a concern. Moreover, the heterogeneous structure has been found as an intriguing approach to improve the on-current of CNTTFET with carbon nanotube-GNR heterojunctions [20]. However, the accurate realization of such an atomistic heterojunction is complicated and budget consuming, which is a concern. Therefore, new innovative techniques and simple improvement approaches should be developed while considering the fabrication aspect and TFET performance.

In this computational work, an efficient approach based on the synergy of electrostatic and chemical-doping engineering is proposed to boost the subthreshold and switching performance of sub–10 nm junctionless-carbon-nanotube-tunnel field-effect transistors. The proposed viable approach has been found to be very efficient in shrinking the band-to-band tunneling (BTBT) window and dilating the DSDT barrier, while boosting the subthreshold and switching performance of ultrascaled JL CNTTFET. The improved characteristics include the on-current, off-current, current ratio, subthreshold swing, leakage current, ambipolar behavior, I_60_ factor, power-delay product, and intrinsic delay. The proposed design has also shown high-performance in ultrascaled regime (with 5-nm gate length), where the sub-thermionic SS and high current ratio have been within reach.

The rest of this article is structured as follows. Section 2 details the proposed TFET structure. Section 3 summarizes the quantum simulation approach. Section 4 is devoted to present and discuss the results. Section 5 contains the conclusion.

## 2. Device Structure

**Figure 1:**
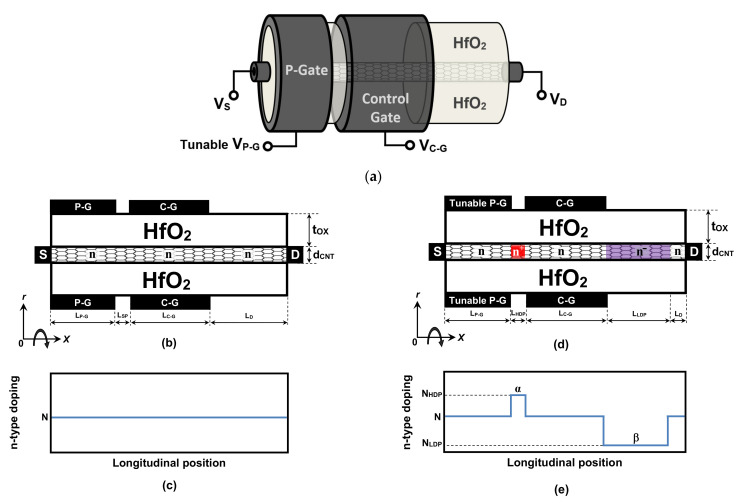
(**a**) Three-dimensional structure of the JL CNT tunneling FET. (**b**) Lengthwise-cut view and (**c**) doping profile of the conventional uniformly n-type doped JL CNT tunnel FET. (**d**) Lengthwise-cut view and (**e**) doping profile of the proposed non-uniformly n-type doped JL CNT tunnel FET with tunable P-G voltage.

Figure 1a shows the three-dimensional (3D) structure of the junctionless-carbon-nanotube-tunneling field-effect transistor (JL CNTTFET). The shape of the nanodevice follows the cylindricity of the carbon-nanotube channel, and, thus, coaxial gates are considered accordingly. Note that the gate-all-around (GAA) configuration is found to be more efficient in terms of controlling the carrier transport [21,22]. In addition, the GAA structure really supports the assumption of uniform electrostatics in radial direction, thus making the simulation less complex [22]. In this work, a small CNT diameter was used, due to its appropriateness in terms of device electrical performance. Figure 1b shows the lengthwise-cut view of the uniformly doped JL CNTTFET. As shown, the tunneling FET is endowed with an auxiliary gate (P-gate) to electrostatically p-type dope the source side in order to preserve the junctionless aspect on CNT channel while achieving the tunneling FET operating regime [9]. As we can see, the control-gate at the middle of the device governs the FET carrier transport, while the drain side is left undoped. The Hafnium oxide (HfO_2_) is considered to be a gate dielectric surrounding the zigzag CNT (Z-CNT). Figure 1c shows the doping profile of the conventional JL CNTTFET, which is uniformly n-type doped from source to drain electrodes. Figure 1d shows the cross-sectional view of the proposed engineered doping (ED)-based design, EDJL CNTTFET. As shown, this latter design is similar to the baseline design shown in Figure 1b, with the exception of three differences. The first is a heavily n-type doped pocket (HDP), which is located between the two coaxial gates with *α* concentration [23], the second is a lightly n-type doped portion (LDP) near the drain with *β* concentration, and the third is an electrical p-type doping gate with a tunable applied bias that aims to match the synergy. Figure 1e shows the doping profile of the proposed JL CNTTFET, showing the heavily n-type doped pocket between the P-G and C-G gates and the LDP near the drain electrode. From a fabrication point of view, the proposed non-uniform doping profile can be reached by tuning the exposure time of the concerned CNT portions to the employed chemical dopant and thus varying the doping level as required [13,22].

All physical, electrical, and geometrical design parameters are shown in Table 1. Note that the physical backgrounds and reasons for the adopted chemical and electrical doping and their locations are discussed thoroughly in Section 4.

## 3. Simulation Approach

In the literature, the common quantum simulation method used to propose, investigate, and assess advanced nanoscale CNTFETs with full soundness and high accuracy is the self-consistent computation between the non-equilibrium Green’s function formalism and the Poisson equation [22,23,24,25]. The main assets of this quantum simulation method are its ability to consider most of electrostatic features and the main quantum transport phenomena, including the band-to-band and direct source-to-drain tunneling mechanisms [22,23,24,25,26]. For this reason, we adopted the NEGF simulation in the present computational work. The retarded Green’s function is the main equation on which this quantum simulation is based, and it can be expressed in the following matrix form [26]

(1) $$G(E)={\left[(E+i\eta^{+})I-H_{PZ}-\Sigma_{S}-\Sigma_{D}\right]}^{-1}$$

where *E*, *η*^+^, *H_PZ_*, *I*, and Σ*_S_*_(*D*)_ are the energy, infinitesimal positive value, Hamiltonian matrix based on the atomistic nearest neighbor *p_Z_*-orbital tight-binding approximation, identity matrix, and the source (drain) self-energy, respectively. In our computation, the mode space (MS) representation is employed to avoid the computational burden while considering only the relevant modes and the ballistic limit conditions [27]. Note that the source (drain) self-energy is analytically computed in accordance with the MS computational fashion [22,27]. The computation of the retarded Green’s function and the S/D self-energies allows us to compute the source (drain) local density of states (LDOS), *D_S(D)_*, using the following expressions [22]

*D_S(D)_* = *GΓ_S(D)_G*^†^ (2)

with

(3) $$\Gamma_{S(D)}=i(\Sigma_{S(D)}-\Sigma_{S(D)}^{†})$$

where Γ*_S_*_(*D*)_ denotes the energy level broadening due to the S/D contact. Now, the channel charge density is within reach, using the following equation [22]:

(4) $$\begin{array}{l} Q(x)=(-q)\int_{-\infty }^{+\infty }dE\;\cdot \;\mathrm{sgn}\left[E-E_{N}(x)\right]\times \left\{D_{S}(E,x)\right.f(\mathrm{sgn}\left[E-E_{N}(x)\right](E-E_{FS}))\\ \;\;\;\;\;\;\;\;\;\;\;\;\;\;\;+D_{D}(E,x)f(\mathrm{sgn}\left[E-E_{N}(x)\right](E-E_{FD})\left.)\right\} \end{array}$$

where *q*, *sgn*, *E_N_*, *f*, and *E_FS_*_(*FD*)_ are the electron charge, sign function, charge neutrality level, Fermi function, and S/D Fermi level, respectively. In the self-consistent computation, computing the charge-density Equations (1)–(4) needs information on the on-site electrostatic potential, which is approximated by solving the Poisson equation for cylindrical nano-FET structure given by the following equation [22,27]:

(5) $$\nabla^{2}U(x,r)=-\frac{\rho (x,r)}{\varepsilon }$$

where *U*, *ε,* and *ρ* are the potential distribution, the dielectric constant, and the Z-CNT charge density, including the chemical doping concentration, respectively. The Poisson equation is solved by using the finite difference method, while assuming that the potential is invariant in the coaxial direction. The Dirichlet boundary conditions are imposed on the gates’ nodes, considering the relevant biases, while the Neumann boundary conditions are considered for the remaining external interfaces, including the source and drain electrodes [22,27]. After attaining the self-consistency between the Poisson solver and the MS NEGF solver, the drain current is within reach by using the following equation [22]:

(6) $$I=\frac{4q}{\hbar }\int_{-\infty }^{+\infty }dE\;\;T(E)\;[f(E-E_{FS})-f(E-E_{FD})]$$

where *ħ* is the Planck’s constant, and *T*(*E*) is the transmission coefficient, which can be computed as follows [22]:

(7) $$T(E)=Tr\;[\Gamma_{S}G\Gamma_{D}G^{†}]$$

where *Tr* denotes the trace operator. All NEGF simulations were performed by using MATLAB software. For more information and details regarding the NEGF-based quantum mechanical simulation of nanoscale carbon-nanotube FETs, we refer to our previous relevant works [24,25,28,29], where the validation of the used NEGF simulation against some experimental and theoretical data was reported. 

## 4. Results and Discussion

**Figure 2:**
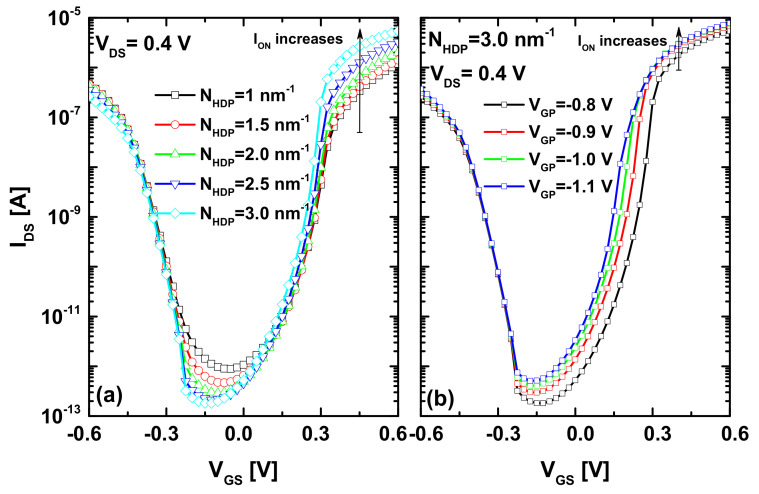
(**a**) Impact of doping concentration of the heavily n-type doped pocket between gates on the I_DS_–V_GS_ transfer characteristics of JL CNTTFET. (**b**) Impact of P-gate voltage on the improved I_DS_–V_GS_ transfer characteristics of JL CNTTFET endowed with HDP.

The nanoscale tunneling FETs are promising nanodevices, due to their assets, namely sub-thermionic subthreshold swing, low-off current, and intriguing scaling capability. However, the low on-current is considered the main disadvantage in these promising nano-FETs. Thereafter, we show interesting improvements in on-current, off-current, and subthreshold swing, using the synergy of both chemical and electrical-doping techniques, while keeping the junctionless paradigm. Figure 2a shows how the increase in doping concentration of the heavily n-type doped pocket boosts the on-current of the JL CNTTFET. When inspecting the same figure, we can observe that the off-current is also slightly improved with the N_HDP_ increase. The recorded off-current (on-current) improvement is principally attributed to the dilation (shrinking) in the DSDT (BTBT) window induced by the heavily doped pocket. Figure 2b shows that the recorded improvement in on-current, using the heavily doped pocket, can be further enhanced by increasing negatively the applied voltage of the auxiliary p-gate that ensures the source p-type doping electrostatically. We can also see that a slight increase in off-current is recorded, while the ambipolar behavior is still the same. The recorded additional improvement in on-current is logically attributed to an additional shrinking in the BTBT window that is induced by the negatively high p-gate voltage. Therefore, in order to increasingly boost the on-current, it is appropriate to combine the HDP technique with that of the negatively high p-gate voltage.

**Figure 3:**
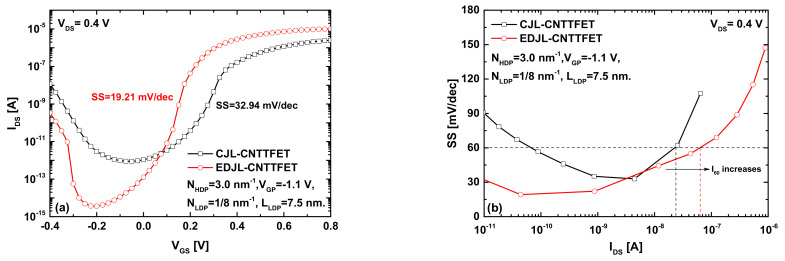
(**a**) I_DS_–V_GS_ transfer characteristics of the conventional JL CNTTFET and the proposed JL CNTTFET, which includes the heavily n-type doped pocket, the optimized p-gate voltage, and the LDP near the drain electrode. (**b**) Subthreshold swing as a function of drain current for the standard and proposed nano-TFET.

In order to decrease the off-current (increased with increasing the negative p-gate voltage as shown in Figure 2b) and improve the subthreshold swing, we adopted, in addition, a lightly doped portion to dilate the direct source-to-drain tunneling window, while keeping the junctionless paradigm. As expected, Figure 3a explicitly shows significant improvements in terms of on-current, off-current, subthreshold swing, and ambipolar behavior, in comparison to the conventional JL CNTTFET. We can clearly see the steep switching of the transfer characteristic, which is a highly desired feature in cutting-edge high-performance digital applications. Figure 3b shows the subthreshold swing in function with the drain current for the conventional and proposed nanoscale TFETs. This indicates that the drawn curves are important and informative, because they reveal the minimum SS on the one hand and the values of SS over the transfer characteristics on the other hand. The same figure also highlights the I_60_ factor, which denotes the highest drain current at which SS = 60 mV/dec is recorded. Note that the ideal region of the I_60_ metric in the plot is on the lower right corner, with a steep SS and high drain currents [30]. As shown, the performance of the proposed design is closest to the aforementioned region of interest, with a higher I_60_ factor in comparison to the baseline TFET. In addition, the proposed JL-CNTTFET exhibits a steeper SS than the conventional TFET over the considered I_DS_ range, and, thus, the average SS of the proposed JL-CNTTFET is smaller than that of the conventional one. It is worth noting that the proposed (conventional) design provides a minimum SS value of ~19 mV/dec (~33 mV/dec), as indicated in Figure 3a.

**Figure 4:**
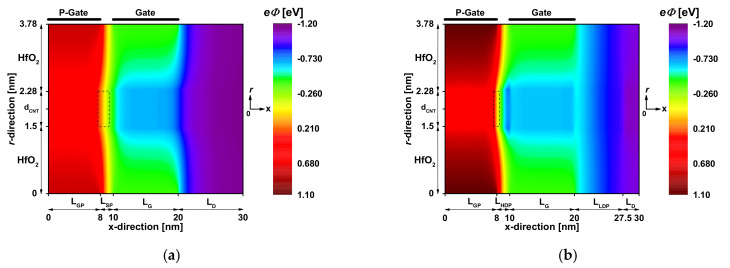
Two-dimensional potential distribution at V_GS_ = 0.4 V and V_DS_ = 0.4 V for (**a**) the CJL CNTTFET and (**b**) the proposed JL CNTTFET with chemical- and electrical-doping engineering.

Figure 4 shows the potential distribution drawn from the converged Poisson’s solutions at the lengthwise-cut region. The electrostatic gating of the p-gate and the main gate is clearly seen. More important, we can see in Figure 4a that the longitudinal potential variation between the two aforementioned electrostatic-gating examples (at the level of the ungated region, framed by a discontinued line) is somewhat wide, while reflecting the long BTBT window responsible for the low on-current. However, by using the V_PG_ adjustment and heavily n-type doped pocket, we can observe a steep longitudinal potential variation at the BTBT region, as shown in Figure 4b. In this latter example, it is also clearly seen the dilation in the DSDT window that is induced by the lightly n-type doped portion near the drain, making the nano-TFET more immune to the DSDT leakage, contrary to the conventional case.

**Figure 5:**
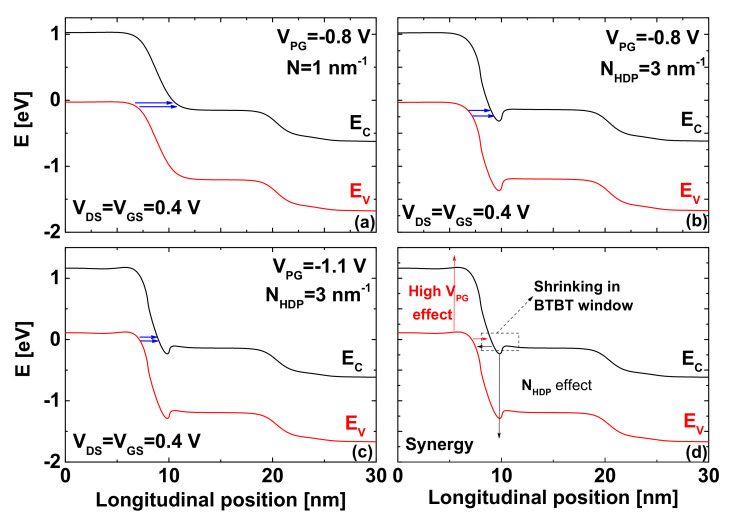
Band diagrams drawn at the on-state condition of (**a**) CJL CNTTFET, (**b**) JL CNTTFET with heavily n-type doped pocket, and (**c**) JL CNTTFET with both highly n-type doped pocket and optimized P-G voltage. (**d**) Doping-induced shrinking in BTBT window.

Figure 5 shows how the band diagrams are tuned by using the chemical- and electrical-doping techniques in order to improve the low on-current, which is among the main drawbacks in nanoscale TFETs. In Figure 5, the top (bottom) solid line is the edge of conduction (valence) band edge, E_C_ (E_V_). We can clearly see in all figures that the edge of the conduction band underneath the gate is below the edge of the source valence band, while allowing a band-to-band tunneling mechanism that results in the on-current in tunneling FETs. This indicates that the direct source-to-drain tunneling can also contribute to the BTBT on-current, especially in TFET with ultra-scaled gate lengths, where the DSDT leakage becomes a concern. In Figure 5a, we can clearly see that the BTBT window indicated by two arrows is somewhat long, leading to low TFET on-currents. In Figure 5b, we can see the HDP-induced band lowering, which shrinks the BTBT window while increasing the BTBT components and making the on-current higher, as shown in Figure 2a. It is worth noting that the shorter (longer) BTBT window provides a higher (lower) on-current [18]. The inspection of Figure 5b also reveals a slight dilation in the DSDT window, due to the HDP-induced band lowering, while also explaining the recorded decrease in off-current shown in Figure 2a. Figure 5c shows that the BTBT window becomes somewhat shorter by increasing the p-gate voltage, and, thus, the BTBT on-current is boosted accordingly. Note that the negative increase in p-gate voltage also decreases the DSDT window, leading to the increase in off-current, as recorded in Figure 2b. For more clarification, Figure 5d is plotted to graphically show how the synergy of the HDP-based technique and the P-G voltage adjustment increasingly shrinks the BTBT window responsible for the on-current increase. In fact, the negative increase in P-gate voltage induces a band elevation at the level of source region, as shown in the same figure. Therefore, geometrically, the V_PG_ adjustment-induced band elevation, together with the HDP-induced band lowering, shrinks the BTBT window more and more, making it shorter, while clearly explaining the additional increase in on-current recorded in Figure 2b.

**Figure 6:**
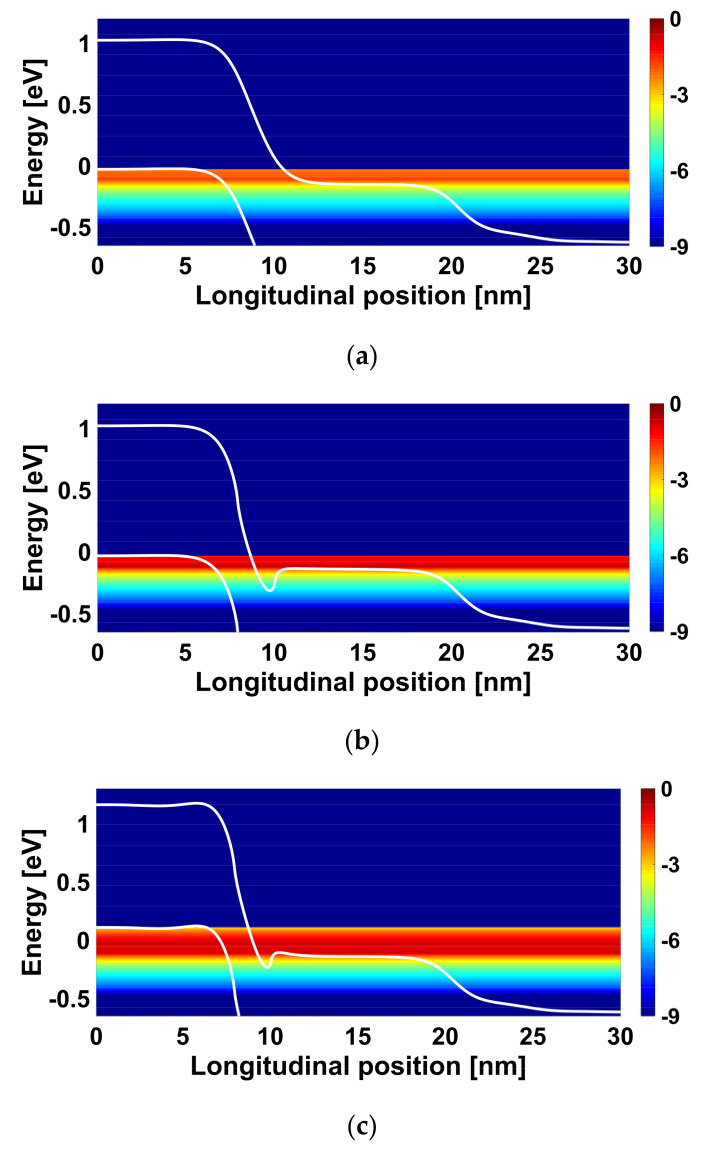
Energy-position-resolved current spectrum at on-state (V_GS_ = 0.4 V and V_DS_ = 0.4 V) of (**a**) the CJL CNTTFET, (**b**) the JL CNTTFET with heavily n-type doped pocket, and (**c**) JL CNTTFET with both HDP (N_HDP_ = 3 nm^−1^) and optimized p-gate bias (V_PG_ = −1.1 V).

Figure 6 shows the energy-position-resolved current spectrum drawn from the NEGF quantities for the JL CNTTFETs under investigation. We can see in Figure 6a the band-to-band tunneling from source valence band to the drain conduction band through the BTBT window. In Figure 6b, we can clearly see that the BTBT on-current spectrum becomes higher than that of conventional JL CNTTFET, due to the HDP-induced band lowering that shrinks the BTBT window. Figure 6c obviously shows that the synergy of the p-gate voltage adjustment and heavily doped pocket approaches causes an additional increase in BTBT on-current spectrum (in comparison to other cases), due to the recorded additional shortening in BTBT window, as previously explained and shown in Figure 5.

**Figure 7:**
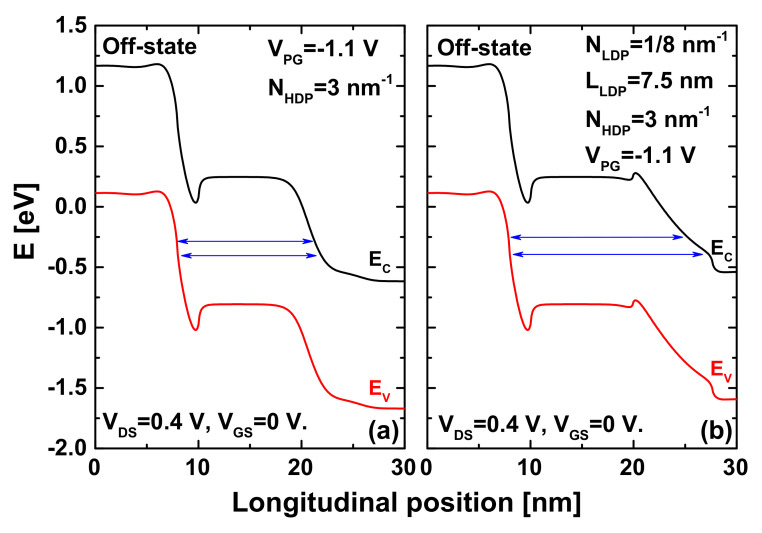
Band diagrams at the off-state for (**a**) JL CNTTFET with HDP and optimized V_PG_; and (**b**) JL CNTTFET endowed with HDP, optimized V_PG_, and lightly n-type doped ZCNT portion near the drain electrode.

Figure 7 shows the role of the lightly n-type doped ZCNT region near the drain in dilating the direct source-to-drain tunneling window responsible for the tunneling leakage current in ultrascaled TFETs. As shown in Figure 7a, the DSDT window of the JL CNTTFET without the lightly n-type doped pocket is somewhat short (~14 nm), leading to a higher leakage current or, equivalently, a higher DSDT current; thus, a high off-state is recorded, as shown previously in Figure 2. Figure 7b clearly shows the LDP-induced dilation in the DSDT window by elevating the concerned bands via the lightly doped pocket. Please note that this LDP-induced dilation in the DSDT window explains the recorded improvement well in the off-current, I_60_ factor, and sub-thermionic subthreshold swing, as shown above in Figure 3.

**Figure 8:**
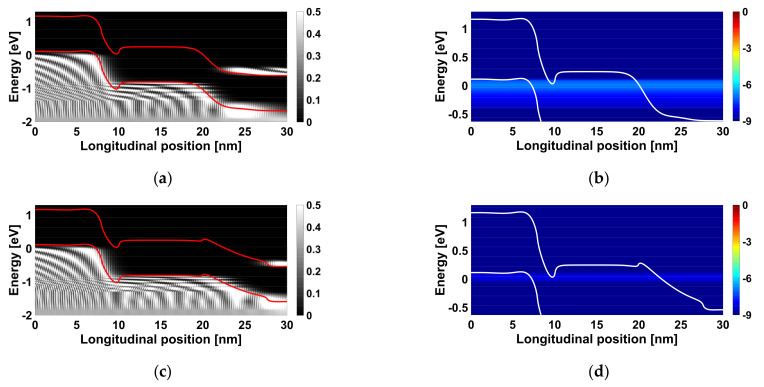
Electron density per unit energy versus the longitudinal position at off-state (V_GS_ = 0 V and V_DS_ = 0.4 V) for the JL CNTTFET (**a**) without and (**c**) with (L_LPD_ = 7.5 nm and N_LDP_ = 1/8 nm^−1^) the lightly n-type doped portion near the drain electrode. Energy-position-resolved current spectrum for the JL CNTTFET (**b**) without and (**d**) with (L_LPD_ = 7.5 nm and N_LDP_ = 1/8 nm^−1^) the LDP near the drain electrode.

Figure 8a shows the electron-density distribution throughout the JL CNTTFET, without considering the lightly n-type doped pocket near the drain electrode. We can see that the direct source-to-drain tunneling window is somewhat short; equivalently, the source and drain reservoir are close, thus leading to a significant DSDT mechanism and a high leakage current spectrum, as shown in Figure 8b. Figure 8c shows the electron density per unit energy versus the longitudinal position at off-state for the JL CNTTFET, considering the LDP near the drain electrode. As shown, the source and drain reservoirs diverge, making the DSDT window longer, and, thus, a decrease in DSDT off-current spectrum is recorded, as shown in Figure 8d.

**Figure 9:**
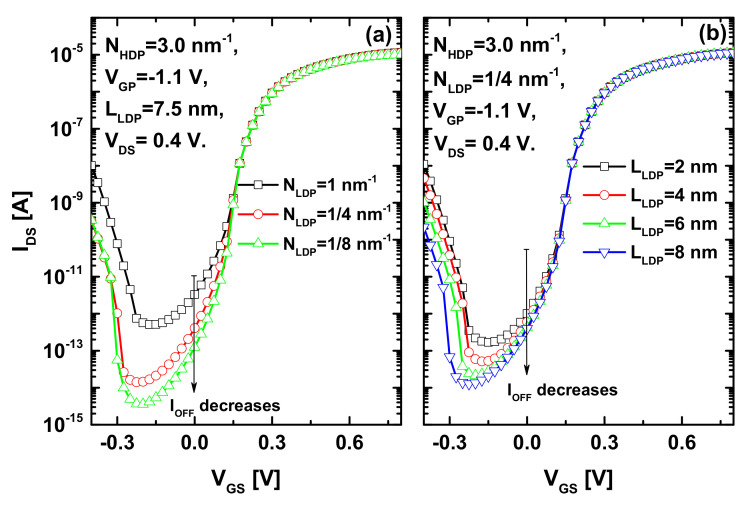
Impact of (**a**) doping concentration of the lightly n-type doped pocket near the drain and (**b**) the length of LDP on the I_DS_–V_GS_ transfer characteristics of the proposed JL CNTTFET.

Figure 9a shows how the decrease in doping concentration of the lightly n-type doped pocket near the drain improves the subthreshold swing and off-current and suppresses the ambipolar behavior. It is worth noting that we have not considered very low doping concentrations in order to keep the junctionless paradigm and avoid the n-type doping-intrinsic abrupt junction. The recorded improvements are attributed to the light doping-induced band elevation that dilates the DSDT window. In Figure 9b, the same improvement behavior is recorded when increasing the length of the LDP, where enhancements in terms of sub-thermionic subthreshold swing, off-current, and ambipolar behavior are recorded, while optimized on-current is within reach by the chemical and electrical-doping techniques near the source, as shown above. Therefore, wide lightly n-type doped ZCNT portions with a low concentration are suitable for improved subthreshold performance; however, there are some considerations regarding the junctionless aspect, the scaling capability, and the ohmic drain contact.

**Figure 10:**
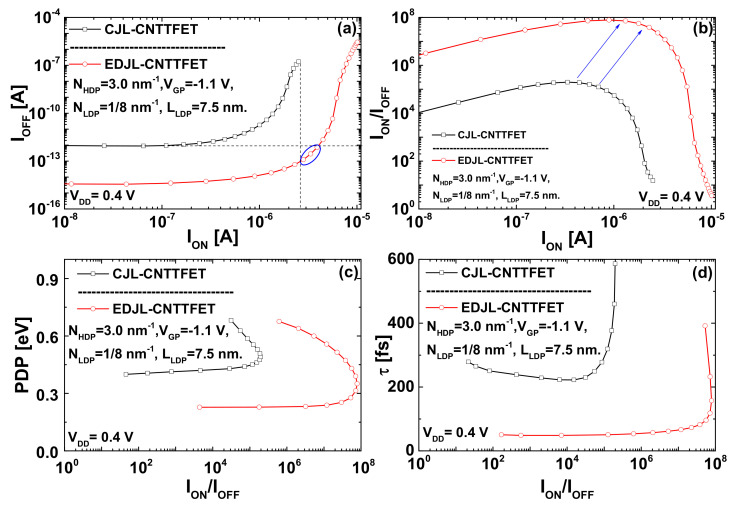
(**a**) Off-current versus on-current, (**b**) I_ON_/I_OFF_ current ratio in function of the on-current, (**c**) power delay product as a function of current ratio, and (**d**) intrinsic delay versus the I_ON_/I_OFF_ current ratio for the conventional and proposed JL CNTTFETs.

Figure 10 shows the switching performance of the conventional and proposed nanoscale TFETs, including the off-current (*I_OFF_*); on-current (*I_ON_*); current ratio (*I_ON_*/*I_OFF_*); power-delay product, *PDP* = *(Q_ON_* − *Q_OFF_)V_DD_*; and the intrinsic delay, *τ* = *(Q_ON_* − *Q_OFF_)/I_ON_*. It is to indicate that the intrinsic delay presents how fast the JL CNTTFET can switch, while the power-delay product shows the energy required for a switching event. Note that the curves in Figure 10 are drawn from the concerned transfer characteristics by shifting a switching window with a width of power-supply voltage (*V_DD_*) equal to 0.4 V, while extracting the on-state total charge (*Q_ON_*) and its current (*I_ON_*) at each given *V_GS-ON_*, and the corresponding off-state total charge (*Q_OFF_*) and its current (*I_OFF_*) at *V_GS-OFF_ = V_GS-ON_ − V_DD_* [31,32,33].

Figure 10a shows that the proposed JL CNTTFET can provides higher (lower) on-current (off-current) for a shared off-current (on-current) in comparison with the conventional nanodevice. Our inspection of the same figure reveals that the proposed JL CNTTFET, which is endowed with electrical- and chemical-doping engineering, can provide a particular performance (highlighted by a solid circle), where both higher on-current and lower off-current were simultaneously recorded in comparison with the currents of the conventional JL CNTTFET. Figure 10b is drawn from the concerned transfer characteristics, showing that the proposed JL CNTTFET can exhibit a higher maximum reachable current ratio (MRCR) with higher on-current, as indicated by arrows. Note that the MRCR of the proposed device is higher than that of the conventional device by about three orders of magnitude. In addition, we can clearly see that the proposed device exhibits a higher I_ON_/I_OFF_ current ratio than the conventional device over the shared range of on-currents. Figure 10c shows and compares the power-delay product (PDP) in function of I_ON_/I_OFF_ current ratio for the proposed and conventional JL CNTTFET. It is clearly seen that the proposed nanodevice exhibits lower PDP (higher I_ON_/I_OFF_) than its conventional counterpart over the shared range of current ratio (PDP). In addition, we can observe that the proposed device exhibits a higher MRCR with a lower PDP than that of the CJL CNTTFET. The recorded improvements in terms of PDP empower the proposed design to be an intriguing energy-efficient nano-TFET for high switching applications. Figure 10d shows that the proposed device provides faster (higher) intrinsic delay (current ratio) than its conventional counterpart over the shared range of current ratio (intrinsic delay). In addition, we can also see that the proposed design provides higher MRCR with faster delay than that of the CJL CNTTFET. The substantial decrease in terms of intrinsic delay, together with the recorded current ratios, makes the proposed JL CNTTFET an interesting nanoscale junctionless tunnel FET for high-speed applications. 

In order to assess the benefits of the proposed design in the ultrascaled regime, we have performed a quantum-simulation-based comparison between the conventional and the proposed nanodevices, considering the main parameters of switching performance. Table 2 summarizes the main switching figures of merit of the proposed JLCNTTFET with 5 nm gate length. As very interesting results, the current ratio is improved by about 3 orders of magnitude and sub-thermionic SS (43 mV/dec) is well recorded in ultra-scaled regime. In addition, the on-current is boosted, and the off-current, minimum leakage current (I_MIN_), I_60_ factor, PDP, and intrinsic delay are all decreased, which is very important for high-speed, low-power, and high-performance switching applications. 

**Figure 11:**
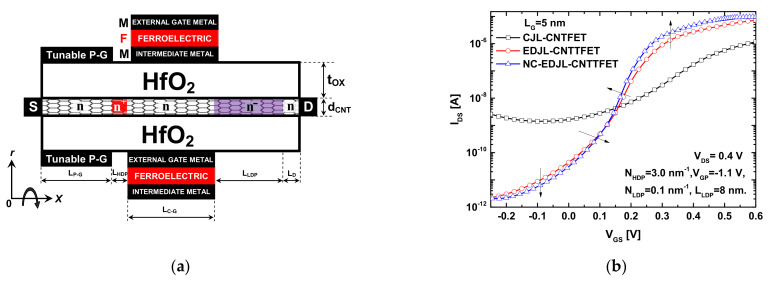
(**a**) Cross-sectional view of the proposed NC-EDJL-CNTTFET with MFMIS structure. (**b**) I_DS_–V_GS_ transfer characteristics of the conventional JL CNTTFET, the proposed EDJL-CNTTFET, and the proposed NC-EDJL-CNTTFET considering 5 nm gate length.

Basing on the recorded results in terms of the on-current improvement, which is attributed to the doping-induced shrinking in BTBT window, the ferroelectric-based gating can be adopted as additional improvement approach in order to further improve the EDJL-CNTTFET performance via the feature of the FE-induced amplified gate voltage [34], and thus well exploiting the boosted BTBT on-current. In fact, the adoption of ferroelectric (FE) material can take two different designs. The first configuration is based on the metal–ferroelectric–insulator–semiconductor (MFIS) design, while the second arrangement is the metal–ferroelectric–metal–insulator–semiconductor (MFMIS) structure [35]. We adopt in our case the MFMIS configuration due to its benefits in terms of elaboration [35,36,37], the possibility of separate integration [38], and the simulation simplicity [39,40,41]. Figure 11a shows an EDJL-CNTTFET design with a MFMIS structure. Note that the MFMIS can be integrated as a coaxial gate [42] or used as separate gating system ideally connected by a wire [25,38,41,43]. From simulation point of view, the ferroelectric field-effect transistors endowed with a MFMIS system can be treated as a baseline field-effect transistor in series with a ferroelectric capacitor [25,40,41,42,43,44]. Therefore, conceptually, the numerical modeling of the negative capacitance (MFMIS) nanodevices is divided into two parts [45]. The first step of simulation deals with the baseline device as mentioned above in the Section 3. After the self-consistency, the gate charge (*Q_G_*) is numerically extracted and used to compute the voltage across the FE material (*V_FE_*), using the 1-D steady-state Landau–Khalatnikov equation, which is given as follows [34]:

*V_FE_* = 2*αt_FE_Q_G_* + 4*βt_FE_Q_G_*^3^ + 6*γt_FE_Q_G_*^5^ (8)

where *t_FE_* is the FE thickness; and (α, β, and γ) are the FE Landau coefficients, which are chosen to be as those of the Al-doped HfO_2_ FE parameters [25,44,45,46]. After computing *V_FE_*, the external gate voltage (*V_GS_*) of the EDJL-CNTTFET is normally computed by using the following equation [25,39,40,41,42,43,44,45]:

*V_GS_* = *V_INT_* + *V_FE_* (9)

where *V_INT_* is the internal metal-gate voltage considered in the baseline self-consistent quantum simulation. For more computational information regarding the quantum simulation of ultrascaled MFMIS FE-FETs, we refer the reader to our previous works [19,25,45].

Figure 11b shows that the proposed electrical- and chemical-doping approach can significantly improve the *I_DS_*–*V_GS_* transfer characteristics of an ultrascaled JL CNTTFET with 5 nm gate length. We can clearly see the substantial improvements in terms of *I_ON_*, *I_OFF_*, current ratio, and leakage current. In addition, we can observe that the nanodevice with the MFMIS structure additionally improves the on-current, off-current, and subthreshold swing, due to the FE-induced amplified gate voltage. Note that the recorded sub-thermionic subthreshold swing recorded in EDJL-CNTTFET was decreased from 43 to 35 mV/dec via the FE-based improvement approach. This indicates that the adoption of more appropriate FE nanomaterial with particular coercive field and remnant polarization can increasingly boost the nanodevice performance via enhancing the FE-induced amplified internal gate voltage [45]. In order to find the best device and ferroelectric parameters that can lead to the ultimate best performance, a parametric investigation [47] based on metaheuristic techniques (e.g., ant colony optimization, practical swarm optimization, genetic algorithms [48], etc.) in conjunction with the used NEGF simulation approach can be followed, while solving an advanced optimization problem, which can be a matter for future investigations.

The intriguing results obtained in this computational work can give new impulses to the design, simulation, and optimization of the advanced 2D materials-based nanoscale FETs with ultra-thin dielectrics, which have experienced significant progress [49,50,51,52,53,54,55,56,57]. In addition, the employment of such intriguing steep-slope nanodevices in advanced sensing applications [48,58,59,60,61] can be a matter for future works. 

## 5. Conclusions

In this article, a new approach based on the synergy of the electrostatic and chemical-doping engineering is proposed to boost the performance of nanoscale JL CNTTFETs. The hybrid doping approach was found to be efficient at shrinking the BTBT window and dilating the DSDT spacing, while also boosting the JL CNTTFET performance. The profound quantum transport investigations have included the band diagrams, the potential distributions, and the energy-position-resolved electron density and current spectra. As a result, the subthreshold and switching performance is significantly improved, where sub-thermionic subthreshold swing, mitigated ambipolar behavior, boosted on-current, higher current ratio, reduced off- and leakage-current, faster switching speed, lower switching power, and improved scaling capability were obtained. Moreover, the metal–ferroelectric–metal-based gating approach was employed in order to exploit the recorded improvement in carrier transport, while boosting the JL TFET switching performance. The proposed design based on the synergy of electrostatic and chemical-doping engineering solved the main problems in ultrascaled JL CNTTFETs, and this is promising for the future CNT-based nanoelectronics.

## Figures and Tables

**Table 1:** Simulation parameters.

Parameter	Symbol	Value	Unit
Common parameters			
Z-CNT	(n,0)	10	-
Gap energy	E_G_	~1.053	eV
CNT diameter	d_CNT_	~7.82	Å
Gate length	L_C-G_	10	nm
Drain length	L_D_	10	nm
P-gate length	L_P-G_	8	nm
Space between gates	L_SP_	2	nm
S/C/D doping (CJL)	N	1	nm^−1^
Oxide thickness	t_OX_	1.5	nm
HfO_2_ dielectric constant	ε_OX_	16	-
Temperature	T	300	K
Source gate voltage	V_P-G_	−0.8	V
Drain-to-source voltage	V_DS_	0.4	V
Additional parameters in the proposed design			
Heavily doped pocket	N_HDP_	3	nm^−1^
HDP Length	L_HDP_	2	nm
Lightly doped pocket	N_LDP_	N/8	nm^−1^
LDP Length	L_LDP_	7.5	nm

**Table 2:** Switching performance of JL CNTTFETs with 5 nm gate length.

Parameter	CJL-CNTTFET	EDJL-CNTTFET
I_ON_ (A)	7 × 10^−7^	1.34 × 10^−6^
I_OFF_ (A)	3.4 × 10^−9^	1.23 × 10^−11^
I_MIN_ (μA)	1.41 × 10^−3^	2.05 × 10^−6^
I_60_ (A)	-	5.6 × 10^−8^
I_ON_/I_OFF_	205.8	10^5^
SS (mV/dec)	128	43
PDP (eV)	0.52	0.31
τ (fs)	300.8	94.3

## Data Availability

Not applicable.
